# Non-tuberculous Mycobacterium species causing mycobacteriosis in farmed aquatic animals of South Africa

**DOI:** 10.1186/s12866-018-1177-9

**Published:** 2018-04-13

**Authors:** Nomakorinte Gcebe, Anita L. Michel, Tiny Motlatso Hlokwe

**Affiliations:** 1 Tuberculosis Laboratory, Agricultural Research Council - Onderstepoort Veterinary Research, Onderstepoort, South Africa; 2 Department of Veterinary Tropical Diseases, Bovine Tuberculosis and Brucellosis Research Programme, Faculty of Veterinary Science, University of Pretoria, Onderstepoort, South Africa

**Keywords:** Mycobacteriosis, Nontuberculous mycobacteria, Aquatic animals

## Abstract

**Background:**

Mycobacteriosis caused by non-tuberculous mycobacteria (NTM), is among the most chronic diseases of aquatic animals. In addition, fish mycobacteriosis has substantial economic consequences especially in the aquaculture and fisheries industry as infections may significantly decrease production and trade. Some fish NTM pathogens are highly virulent and zoonotic; as such, infection of aquaria with these pathogens is a public health concern.

In this study, we report isolation of nine different NTM species from sixteen aquatic animals including different fish species, frogs and a crocodile. Given the clinical significance of *Mycobacterium marinum* and its close relation to *Mycobacterium tuberculosis,* as well as the significance of ESAT 6 and CFP-10 secretion in mycobacterial virulence, we analysed the *esxA* and *esxB* nucleotide sequences of *M. marinum* isolates identified in this study as well as other mycobacteria in the public databases.

**Results:**

*Mycobacterium shimoidei, Mycobacterium marinum, Mycobacterium chelonae, Mycobacterium septicum /M. peregrinum* and *Mycobacterium porcinum* were isolated from gold fish, Guppy, exotic fish species in South Africa, koi and undefined fish, Knysna seahorse, as well Natal ghost frogs respectively, presenting tuberculosis like granuloma. Other NTM species were isolated from the studied aquatic animals without any visible lesions, and these include *Mycobacterium sp.* N845 T, *Mycobacterium fortuitum*, a member of the *Mycobacterium avium* complex, and *Mycobacterium szulgai*. Phylogenetic analysis of mycobacteria, based on *esxA* and *esxB* genes, separated slow growing from rapidly growing mycobacteria as well as pathogenic from non-pathogenic mycobacteria in some cases.

**Conclusions:**

Isolation of the different NTM species from samples presenting granuloma suggests the significance of these NTM species in causing mycobacteriosis in these aquatic animals. The study also revealed the potential of *esxA* and *esxB* sequences as markers for phylogenetic classification of mycobacteria. Observations regarding use of *esxA* and *esxB* sequences for prediction of potential pathogenicity of mycobacteria warrants further investigation of these two genes in a study employing NTM species with well-defined pathogenicity.

## Background

Non-tuberculous mycobacterial (NTM) infections are among the most common chronic diseases of aquatic animals. NTM are known to infect a number of aquatic animals including fish, amphibians and reptiles [1–5]. NTM infections of fish have been reported in more than 150 species worldwide [6]. The most common pathogens of fish include *Mycobacterium marinum, Mycobacterium fortuitum*, *Mycobacterium chelonae, Mycobacterium peregrinum, Mycobacterium shottsii, Mycobacterium pseudoshottsii* and *Mycobacterium ulcerans* [1, 7–9]. These NTM are of interest as some, particularly *M. marinum* and *M. ulcerans,* are known to be highly virulent and often result in serious outbreaks in freshwater and aquaria animals. Furthermore, infection of fish with these NTM is a public health concern due to their zoonotic nature. *M. marinum* causes the so-called ‘fish finger’s fancier’ or fish tank granuloma, or swimming pool granuloma in humans, depending on where the infection was contracted, while *M. ulcerans* causes Buruli ulcer [10]. *M. marinum* infection in some cases can result in tenosynovitis, arthritis and osteomyelitis [11]. *Mycobacterium shottsii* and *Mycobacterium pseudoshottsii* are both phylogenetically related to *M. marinum* and *M. ulcerans,* and were first isolated from a striped bass (*Morone saxatilis*) [8]. Other NTM like *M. fortuitum*, *M. chelonae* and *M peregrinum* are opportunistic pathogens of fish and humans, especially immuno-compromised individuals [7].

Fisheries and aquaculture remain important sources of food and livelihood for millions of people worldwide. International trade also plays huge socio-economic roles in the aquaculture and fisheries industries, as food security contributor, employment creator, income generator as well as economic growth and development. The aquaculture fish trade industry is reported to have expanded considerably in recent decades [12]. Likewise, reports of mycobacterial infection are on the rise [4, 8, 13–17]. Contrary to other African countries like Nigeria and Uganda, the aquaculture industry in South Africa is still at its infancy. As such, the state has identified this industry as one of the critical areas for promotion. Fish diseases may have substantial economic consequences in the aquaculture industry as infections may significantly decrease production and trade. Control of mycobacterioses in aquaria is by destruction of affected stock and disinfection of the environment as there is no widely used treatment for mycobacterioses in fish [7]. Antibiotic treatment may be a possibility, but is rarely used in the aquaria. The choice of antibiotic is dependent on the infecting NTM species and the strain type [7]. Therefore, monitoring as well as NTM speciation and strain characterization is important in the design of treatment regimens for aquaria mycobacterioses. Currently, in South Africa, limited data is available concerning NTM species circulating in the country’s emerging aquaculture industry, let alone the genotypes of each NTM pathogen that may threaten this industry. Isolation of *M. fortuitum* from three fish species originating from fresh water ponds in South Africa was reported in 1990 [7]. In addition, two *M. marinum* isolates originating from two *Anableps anableps* (four eyed fish) were used together with isolates from other countries in a study to investigate the genetic diversity and population structure of *M. marinum* [11].

Virulence profiling is also an important characteristic that may aid in designing improved control measures including development of effective vaccines and drugs. The most studied virulence associated attribute in pathogenic mycobacteria is the ability of the pathogens to subvert host immune defenses characterized by secretion of the components of a specialized system called the type VII secretion system [18]. The mycobacterial type VII secretion system consists of five ESX system (ESX-1 to ESX-5) which were all most likely duplicated from ESX-4 system [19]. ESX-1, ESX-3 and ESX-5 systems have been shown to be involved in protein secretion and are important for virulence of *Mycobacterium tuberculosis* [20]. The ESX-1 secretion system encoding the region of difference 1 (RD1) (Rv3871-Rv3879c) is the most studied. ESAT 6 (early secretory antigenic target -6kDA) and CFP-10 (culture filtrate protein) encoded by *esxA* (Rv3875) and *esxB* (Rv 3874), respectively, have been the focus of research for development of vaccines and immunological tests for the diagnosis of tuberculosis in both humans and animals. Several studies have shown that secretion of ESAT 6 and CFP-10 is required for the RD-1 mediated virulence in *M. tuberculosis* [21, 22]*.*

In this study, we report isolation of NTM from different farmed aquatic animals originating from several aquaria in South Africa. We also determined *esxA* and *esxB* gene sequences of *M. marinum* isolates and further investigated the potential use of these genes in phylogenetic classification of mycobacteria as well as in prediction of potential pathogenicity of NTM.

## Results

### Mycobacterium species isolated from aquatic animals

**Fig. 1:**
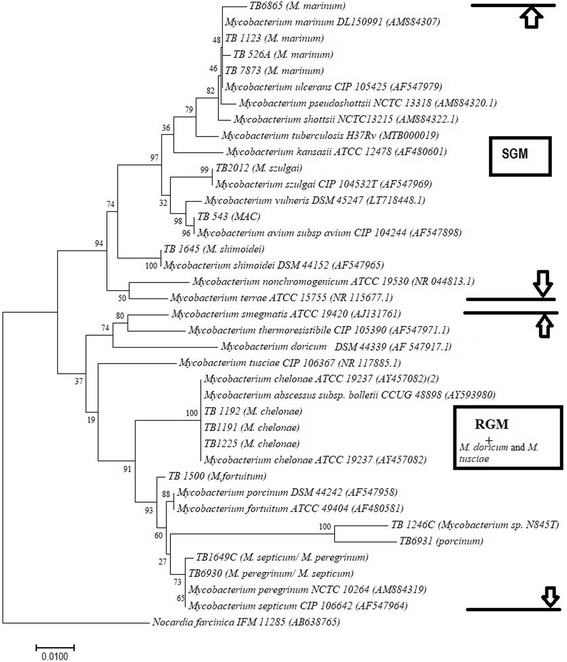
Phylogenetic tree constructed using neighbour joining method, illustrating the genetic position of the isolates from different aquatic species, based on the partial 16S rRNA gene sequences. Genbank accession numbers for the sequences retrieved from the database are shown in parenthesis. The NTM species identified in this study are also shown in parenthesis next to the isolate identity. The percentage of replicate trees (> 50%) in which the associated taxa clustered together in the bootstrap test (1000 replicates) are shown next to the branches (Felsenstein, 1985). *Nocardia farcinica* was used as an out- group sequence. Bar 0.01 substitution per nucleotide

Nine NTM species were identified from 16 samples originating from different farmed aquatic species from marine and fresh water environments (Table 1). *Mycobacterium shimoidei* was isolated from a gold fish (*Carassius auratus*) intestine and ovary presenting granulomous lesions. *Mycobacterium chelonae* was isolated from three Knysna seahorses (*Hippocampus capensis)* originating from the same estuary. One of these seahorses showed a granuloma in the liver and ulceration at the back and under ear, while others did not present any visible lesions. *Mycobacterium* sp. N845 T was also isolated from two seahorses from the same estuary, presenting no lesions. *Mycobacterium fortuitum* was isolated from a Guppy fish (*Poecilia reticulata*) not showing any visible lesions. *Mycobacterium septicum*/ *M. peregrinum* was isolated from a koi fish with no visible lesions and a Natal ghost frog with several large granulomas occurring in visceral fat tissue. *Mycobacterium porcinum* was also isolated from a frog with granuloma in the visceral fat tissue and this frog originated and shared the same aquarium environment as the frog where *M. septicum/ M. peregrinum* was isolated. *Mycobacterium avium* complex species was isolated from an unidentified fish species not presenting any obvious lesions. *Mycobacterium marinum* was isolated from a koi fish, a Guppy fish as well as an exotic fish in South Africa and an unidentified fish species, all showing skin lesions and or ulcerations on the head. *Mycobacterium szulgai* was isolated from a crocodile without any obvious lesions (Table 1). The phylogenetic relatedness, of the NTM species identified in this study and those available on the Genbank database is demonstrated in the neighbor-joining tree based on the 16S rRNA gene in Fig. 1. The *Mycobacterium* species identified in this study clustered together with the respective *Mycobacterium* species available in Genbank.

**Table 1:** NTM species isolated from different aquatic animal species

Isolate ID	NTM species detected	Source of sample from aquatic species		Geographical origin	Environmental origin	Year of NTM isolation	Pathology observed (Granuloma/lesions/mortality)
		Aquatic Species	Organ/sample processed for mycobacterial isolation				
TB1645	*Mycobacterium shimoidei*	Gold fish (*Carassius auratus*)	Whole intestine + ovary	Private aquarium in Pretoria	Fresh water aquarium	1999	Granulomas lesions observed in intestine
TB2012	*M. szulgai*	Crocodile	Lung and heart	Private aquarium in Nylstroom	Fresh water aquarium	2000	No visible lesions observed but mortality occurred
TB6931	*M. septicum/M. peregrinum*	Natal Ghost frog (*Hadromophryne natalensis*)	Whole animal	Aquarium at the National Zoological gardens	Fresh water aquarium	2008	Head lesions
TB1649	*M. septicum/M. peregrinum*	Koi fish	Whole fish	Provincial nature reserve in Western Cape.	Fresh water aquarium	1999	Whole fish, no visible lesions observed, mortality
TB1191	*M. chelonae*	Knysna seahorse (*Hippocampus capensis)*	Whole animal	Estuary in Knysna	Marine water (estuary)	1998	No obvious lesions but mortality occurred
TB1192	*M. chelonae*	Knysna seahorse (*Hippocampus capensis*	Whole animal	Estuary in Knysna	Marine water (estuary)	1998	No obvious lesions but mortality occurred
TB1225	*M. chelonae*	Knysna seahorse (*Hippocampus capensis*)	Whole animal	Estuary in Knysna	Marine water (estuary)	1998	Whole animal with lesions on the liver. White greyish ulceration at the back and under ear.
TB1245	*Mycobacterium* sp. N845 T	Knysna seahorse *(Hippocampus capensis*)	Whole animal	Estuary in Knysna	Marine water (estuary)	1998	No obvious lesions but mortality occurred.
TB1246C	*Mycobacterium* sp. N845 T	Knysna seahorse *(Hippocampus capensis*)	Whole animal	Estuary in Knysna	Marine water (estuary)	1998	No obvious lesions but mortality occurred.
TB1123	*M. marinum*	Koi Fish	Whole animal	Provincial nature reserve	Fresh water aquarium	1998	Lesions and whitish skin ulceration on head.
TB526A	*M. marinum*	Unidentified fish	Whole animal	Private aquarium in Lydenburg	Fresh water aquarium	1995	Lesions on head
TB7873	*M. marinum*	Exotic fish	Whole fish	Private aquarium in KwaZulu Natal	*M. marinum*	Exotic fish	Whole fish
TB6865	*M. marinum*	Guppy fish (*Poecilia reticulate*)	Whole animal	Aquarium at the National Zoological Gardens	Fresh water aquarium	2008	Skin ulceration
TB543	*M. avium subsp. avium/paratuberculosis/hominsuis*	Unidentified fish species	Whole animal	Private aquarium in Lydenburg	Fresh water aquarium	1995	No obvious lesions but mortality occurred
				Private aquarium in KwaZulu Natal	Marine water aquarium	2011	Skin ulceration
TB6930	*M. porcinum*	Natal Ghost Frog (*Hadromophryne natalensis*)	Whole animal	Aquarium at the National Zoological gardens	Fresh water aquarium	2008	Head lesions
TB1500	*M. fortuitum*	Guppy fish (*Poecilia reticulata*)	Whole animal	Aquarium at the National Zoological Gardens	Fresh water aquarium	1998	No obvious lesions, but mortality occurred

### *Mycobacterium esxA* and *esxB* derived phylogeny

**Fig. 2:**
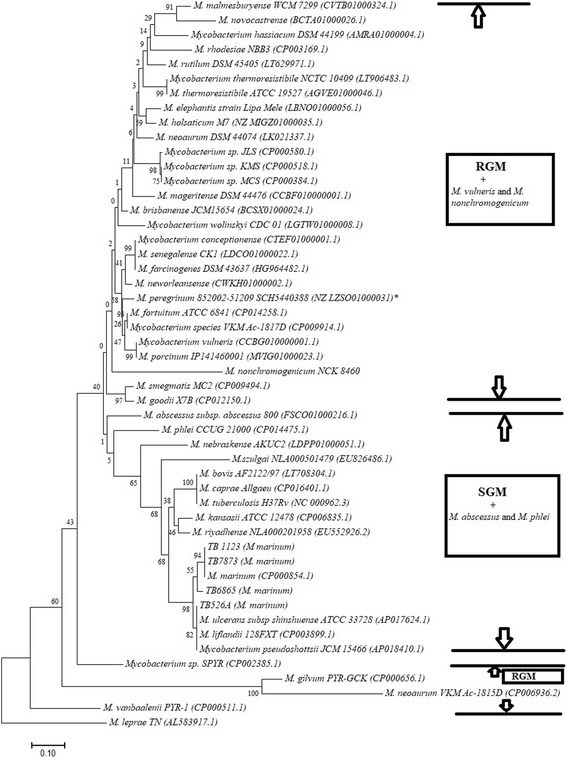
Phylogenetic tree illustrating the evolutionary history was inferred using the Neighbor-Joining method, based on *esxA* gene sequences of *M. marinum* strains identified and other Mycobacteria . The evolutionary distances were computed using the Maximum Composite Likelihood method. Genbank accession numbers for the sequences retrieved from the database are shown in parenthesis. The NTM species identified in this study are also shown in parenthesis next to the isolate identity. The percentage of replicate trees (> 50%) in which the associated taxa clustered together in the bootstrap test (1000 replicates) are shown next to the branches . Evolutionary analyses were conducted in MEGA7 . Bar, 0.1 substitution per nucleotide

**Fig. 3:**
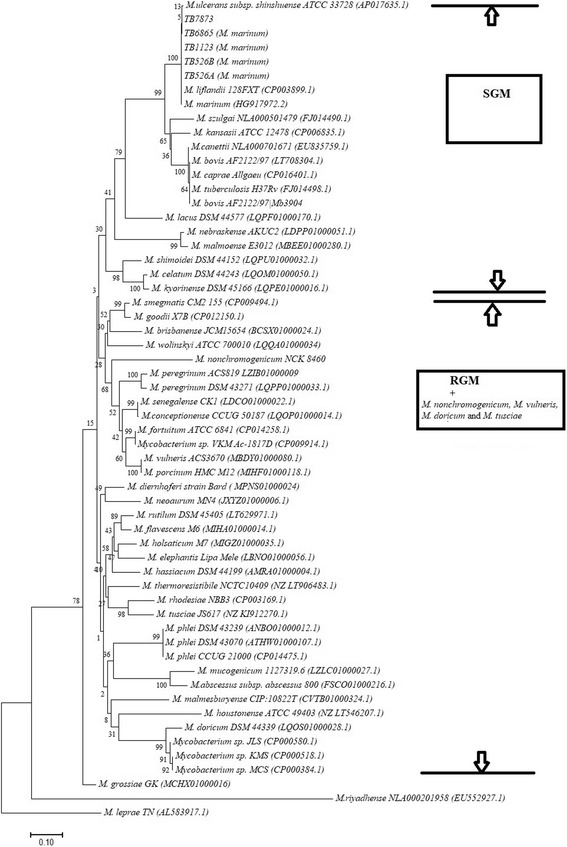
Phylogenetic tree illustrating the evolutionary history was inferred using the Neighbor-Joining method, based on *esxB* gene sequences of *M. marinum* strains identified ana other Mycobacteria . The evolutionary distances were computed using the Maximum Composite Likelihood method. Genbank accession numbers for the sequences retrieved from the database are shown in parenthesis. The NTM species identified in this study are also shown in parenthesis next to the isolate identity. The percentage of replicate trees (> 50%) in which the associated taxa clustered together in the bootstrap test (1000 replicates) are shown next to the branches. Evolutionary analyses were conducted in MEGA7. Bar, 0.1 substitution per nucleotide

Different mycobacterial housekeeping genes, including 16S rRNA, *hsp65, sodA* and the others have been used for species identification and phylogenetic studies. In this study, we inferred the phylogenetic relationship of the *M. marinum* strains identified and those of other *Mycobacterium* species whose *esxA* and *esxB* sequences could be retrieved from Genbank (https://www.ncbi.nlm.nih.gov/nucleotide) in order to investigate the use of these two genes in classifying mycobacteria. *Mycobacterium marinum* strains identified in this study clustered together with *Mycobacterium marinum, Mycobacterium liflandii* and *Mycobacterium ulcerans* from Genbank, as illustrated in the neighbor joining trees in Figs. 2 and 3. Neighbor joining trees generated as illustrated in Figs. 2 and 3 clearly separated the slow-growing mycobacteria (SGM) from the rapidly growing mycobacteria (RGM), with the exception of *Mycobacterium nonchromogenicum* strain NCK 8460, *Mycobacterium vulneris, Mycobacterium tusciae* and *Mycobacterium doricum* that are SGM species, which in this analysis clustered with RGM. Slowly growing NTM species like *M. marinum, M. liflandii, M. ulcerans, M. kansasii, M. riyadhense, M. szulgai* and *M. shimoidei* clustered together with members of the *Mycobacterium tuberculosis* complex (MTBC). The RGM species in these trees formed a separate cluster, containing species that are either not known to cause any diseases or maybe opportunistic pathogens at most. The exceptions to this observation are *Mycobacterium abscessus* subsp. *abscessus* and *Mycobacterium phlei* that are RGM but in the phylogenetic tree based on *esxA* both clustered with SGM*.*

## Discussion

Mycobacteriosis is among the most chronic diseases affecting aquatic animals [3–5, 13]. In this study, sixteen *Mycobacterium* isolates originating from different species of fish, frogs and a crocodile were characterized by 16S rRNA and *hsp65* gene sequence analysis for speciation. All these isolates belonged to nine NTM species namely *Mycobacterium shimoidei, Mycobacterium marinum, Mycobacterium chelonae, Mycobacterium fortuitum, Mycobacterium szulgai, Mycobacterium septicum/ M. peregrinum, Mycobacterium porcinum* and *Mycobacterium* sp. N845 T. *M. shimoidei* was isolated strictly from a gold fish intestine (*Carassius auratus*), originating from a fresh water aquarium, presenting granulomous lesions. *M. shimoidei* was first isolated in 1972 from a patient with a lung infection and since then several other human cases of this NTM have been reported, suggesting that it might be an emerging pathogen of humans [23–25]. To the best of our knowledge, isolation of this NTM from a gold fish presenting mycobacteriosis granuloma is first to be reported in this study. This therefore could potentially be an addition to opportunistic NTM pathogens of fish.

*Mycobacterium marinum* and *Mycobacterium chelonae* are known pathogens of fish [26]. In this study, these NTM species were isolated from different fish species, with *M. chelonae* strictly occurring in sea horses, and *M. marinum* was detected from a Guppy, koi, exotic fish in South Africa and an undefined fish species showing granuloma. The occurrence of *M. marinum* in four different fish species from both fresh and marine water environments, suggested the adaptation of *Mycobacterium marinum* in different water environments and fish species. In South Africa, a case of *M. marinum* infection was last reported in 1987 as Fish tank granuloma [27]. Given its zoonotic nature and its phylogenetic relatedness to *Mycobacterium tuberculosis* complex, it was therefore pertinent that we characterise the *M. marinum* isolates beyond species identification. Virulence profiling is important in the characterisation of microorganisms. Virulence factors in pathogenic mycobacteria are those attributes that enable the bacterium to infect, survive and multiply in host macrophages, resulting in disease symptoms [28]. Albeit the host mounting complex immune responses that often sequesters the pathogen in granuloma, the key to *Mycobacterium* virulence lies in, at least in part with, their ability to establish residence and proliferate inside the macrophages [29]. There is now strong evidence that the ESX-1 secretion system of mycobacteria encoding RD1 is essential for virulence in members of *M. tuberculosis* complex. RD1 mediated virulence is the most studied virulence associated attribute in mycobacteria. Several studies have demonstrated the role of *esxA* and *esxB* in mycobacterial pathogenesis. Some studies have demonstrated an attenuated phenotype in mouse models of infection following inactivation of *esxA* and *esxB* or other genes of the ESX-1 secretion system of *M. tuberculosis* [30–32]*.* These and many other studies thus confirm the significance of ESX-1 secretion system in *Mycobacterium tuberculosis* virulence. Furthermore, some studies have reported loss of ESX-1 secretion, impaired bacterial growth of *M. marinum* in macrophages and reduced bacterial virulence in zebra fish models of infection, resulting from inactivation of either *esxA* or *esxB* as well as transposon mutagenesis in various genes encoded or adjacent the RD1 locus. [33, 34]. Some non-pathogenic NTM, however, harbor *esxA* and *esxB* genes in their genomes with different sequences, whose functions are unknown [18, 35]. For this reason, we set out to investigate, whether sequence differences in *esxA* and *esxB* genes of different mycobacteria could be used in their phylogenetic classification, as well as prediction of their potential pathogenicity. Sequence analysis of *esxA* and *esxB* of the four fish *M. marinum* strains isolated in this study were determined using a PCR- sequencing assay. Phylogenetic analysis of these strains, as well as other NTM that harbor e*sxA* and *esxB,* and members of the *Mycobacterium tuberculosis* complex, revealed phylogenetic profiling that separated SGM, most of which are pathogenic mycobacteria, and RGM which are either not known to be pathogenic or are opportunistic pathogens at most. *Mycobacterium nonchromogenicum* strain NCK 8460, a soil isolate that is SGM [35], and other SGM namely, *Mycobacterium vulneris, Mycobacterium tusciae* and *Mycobacterium doricum* were exceptions to this observation since they were found to be phylogenetically close to the RGM clusters in the *esxA* and *esxB* trees as indicated in Figs. 2 and 3 *M. doricum* and *M. tusciae* were also found to be close to RGM in the phylogenetic analysis based on the 16S rRNA gene as indicated in Fig. 1. Other exceptions were *M. abscessus* subsp. *abscessus* and *M. phlei,* which though they are RGM clustered with SGM species in the *esxA* tree. Therefore, *esxA* and *esxB* may be used as markers for phylogenetic classification of mycobacteria by growth rate to some extent with some exceptions for example *M. nonchromogenicum, M. vulneris, M. tusciae*, *M. doricum, M. phlei* and *M. abscessus* subsp. *abscessus* as seen in this study. We have noted that prediction of pathogenicity of NTM based on *esxA* and *esxB* sequences, as markers, may not result to a definite ruling, as highlighted in this study. We observed that NTM that were previously reported as opportunistic pathogens, were found in some cases to be closer to NTM that are not known to be pathogenic. On the other hand opportunistic RGM pathogens like *M. abscessus* subsp. *abscessus* and *M. phlei* were found to be phylogenetically closer to known pathogenic SGM, suggesting that perhaps in this case *esxA* and *esxB* sequences may be markers for prediction of pathogenicity in NTM. Likewise, some of the non-pathogenic SGM such as *Mycobacterium nonchromogenicum* were found to cluster with non-pathogenic RGM. The established pathogenic NTM, like *M. marinum* and *M. kansasii,* clustered with members of the MTBC, whose pathogenicity is not questioned, in the phylogenetic analysis based on the *esxA* and *esxB* (Figs. 2 and 3). Indeed, observation by van Ingen et al.*,* based on identical sequences of two strains of *M. szulgai* that were clinically relevant or non-relevant, suggested that the gene sequences do not provide a complete picture of pathogenicity in NTM [36]. This suggests that studies that are more extensive on this topic, employing both pathogenic and non-pathogenic *Mycobacterium* species with well-defined pathogenicity are needed to further investigate the association of *esxA* and *esxB* sequence homologies to pathogenicity of mycobacteria. As van Ingen and co-workers previously recommended, description of new species should consider sequence analysis of genes of ESX-1, in particular *esxA* and *esxB*. It should, however, be noted that ESX-1 region is not the only virulence factor in mycobacteria. For instance, some pathogenic members of the *Mycobacterium avium* complex and *Mycobacterium microti* lack this region [19, 37]. However, the potential use of these two genes as markers to predict the potential pathogenic nature of NTM should not be ruled out.

Although *M. chelonae* is a known pathogen of fish, we report its occurrence and its potential role in disease causation in South Africa’s estuary Knysna seahorses for the first time in this study. We also identified *Mycobacterium* sp. N845 T in Knysna seahorses from the same estuary where an outbreak of mycobacterial disease occurred. This is not a validly published *Mycobacterium* species, but it is phylogenetically closely related to *Mycobacterium fortuitum* and *Mycobacterium porcinum* as determined by its partial 16S rRNA gene sequence (Fig. 1)*.* We also identified *Mycobacterium septicum/ M. peregrinum* as well as *Mycobacterium porcinum* each in Natal ghost frogs showing disease symptoms. The two ghost frogs originated from the same fresh water aquarium. A recent study conducted in South Africa reported isolation of *M. abscessus- chelonae* complex from frogs in captivity [5]. Except for a few cases reported in laboratory animals, like those of *Mycobacterium liflandii* and *Mycobacterium marinum,* mycobacteriosis in frogs is rare [38]. *Mycobacterium septicum, M. peregrinum* and *Mycobacterium porcinum* are all known opportunistic pathogens of humans and livestock [26]. Their isolation in amphibians in this study demonstrate the ubiquity of these NTM species as well as their ability to cause disease in a Natal ghost frog. Mycobacteriosis is very rarely reported in crocodiles. In this study, *Mycobacterium szulgai* was isolated from a crocodile sample with tuberculosis like lesions, demonstrating its role in causing disease in aquatic animal. *M. szulgai* is now becoming a common NTM pathogen of humans and has been reported to cause diseases in animals as well [39]. *M. szulgai* infection manifest as both pulmonary and extra pulmonary, although pulmonary disease is the most common manifestation and it may clinically and radiologically resemble pulmonary tuberculosis [40]. As such, isolation of *M. szulgai* is considered clinically significant. *Mycobacterium avium* and *Mycobacterium ulcerans* have been reported to cause generalised granuloma in *Crocodylus johsonii* and *Crocodylus niloticus* respectively [41]. Isolation of a *Mycobacterium avium* complex species from fish demonstrates the ubiquity of these NTM species, which are known opportunistic pathogens of humans and have been isolated from several animal species and the environment.

## Conclusion

In conclusion, we have detected NTM causing mycobacterioses in fish, frogs and a crocodile. Some of these NTM, like *M. marinum* and *M. chelonae* are known pathogens of aquatic animals. Reports of *M. shimoidei* infections in fish are scarce and this NTM is reported for the first time in a gold fish (*Carassius auratus*) in South Africa. This study contributes knowledge about the NTM species causing mycobacterioses in farmed aquatic animals in our country. Even though the study provide limited information regarding NTM diversity in aquatic animals, it does provide insights into the occurrence of pathogenic NTM that may threaten the emerging aquaculture industry in South Africa. Indeed, previous studies conducted country wide to investigate the prevalence of NTM in cattle, buffaloes and other wildlife species, as well as the environment in South Africa, found that NTM are highly abundant and more diverse than in many other parts of the world [42, 43]. This and the previous studies raise serious economic and public health concerns as some NTM isolated have a zoonotic potential, hence, warrants proper monitoring programs of animal mycobacteriosis for control purposes. Sequence differences of *esxA* and *esxB* in different mycobacteria were shown in this study to have a potential use in phylogenetic classification of mycobacteria and in prediction of the potential pathogenicity of NTM species.

## Methods

### Sample origin

Samples used in this study were obtained from routine diagnostic submissions to the Tuberculosis Laboratory of the Agricultural Research Council-Onderstepoort Veterinary Research (ARC-OVR) from different regions of South Africa. Routine submissions at the Tuberculosis laboratory form part of the State Veterinary Service’s strategy for confirming mycobacterial infections in animals. Samples were obtained from the following aquatic animals: crocodile, Natal ghost frog and different species of fish collected during the years 1995–2011. A summary of the animal sample sources including geographic origin, animal species and sample type is presented in Table 1.

### Laboratory examination, *Mycobacterium* isolation and identification

Laboratory examination of the sample to be cultured was performed macroscopically to determine the presence or absence of tuberculous lesions/ granulomas and findings were recorded. Tissue samples as well as whole animals (in case of seahorses, gold fish and frogs) were processed and cultured according to standard laboratory procedures as described by Gcebe and Hlokwe as well as Hlokwe et al.*,* [43, 44]. Ziehl–Neelsen staining was performed to determine the acid fastness of colonies typical of Mycobacterium species, which were selected, based on morphology. The acid-fast isolates were stored at − 20 °C for further molecular analysis.

### DNA extraction

DNA was extracted using the heating method [44]. Briefly, individual acid-fast colonies were suspended in 100 μl of sterile distilled water and heated at 95 °C in a heating block/ or in boiling water for 25 min. The culture lysates were stored at − 20 °C or − 70 °C until further analysis. The culture lysates were used as DNA template in subsequent PCR protocols.

### In vitro *a*mplification and sequencing of the mycobacterial 16S rDNA and *hsp65* genes for speciation

PCR and sequencing of the 577 bp fragment of the mycobacterial 16S rRNA gene was used for mycobacterial speciation [42, 45]. Sequencing of the 439 bp *hsp65* gene fragment was done for differentiation of some NTM species with similar, or identical 16S rRNA, which could not be delineated using the 16S rRNA gene PCR-sequencing assay [43]. Sequence analysis of the *hsp65* gene fragments was performed to differentiate *Mycobacterium chelonae* and *Mycobacterium abcessus* group as well as *Mycobacterium marinum* and *Mycobacterium ulcerans*. Amplification and sequence analysis of both gene fragments were done as described by Gcebe et al.*,* [35].

### Amplification of *esxA* and *esxB* genes of *M. marinum* isolates

Crude DNA extracts were used as template DNA in PCR protocols targeting *esxA* and *esxB* genes. The following primer pairs: esat F: 5’ATGACAGAGCAGCAGTGGAA 3′ and esat R 5’CTATGCGAACATCCCAGTGA3’ located at positions 1–20 and 288–271, respectively, of the *M. marinum esxA* (MMAR_5450) gene sequence were used for amplification of the *esxA* gene. Primers cfp-10F 5’ATGGCAGAGATGAAGACCGA3’ and cfp-10 R 5’ TCAGAAGCCCATTTGCGAGG 3′ located at positions 1–20 and positions 303–284 of the *M. marinum* cfp-10 (MMAR_5449) were used for the in vitro amplification of *esxB* gene sequences. The *M. marinum esxA* and *esxB* gene sequences used to manually design these primers were retrieved from the Marinolist database (http://svitsrv8.epfl.ch/mycobrowser/marinolist.html).

### Phylogenetic analysis of *Mycobacterium* species

To determine the phylogenetic relationship among all the *Mycobacterium* species identified in this study as well as between these species and the other published species, a neighbor joining tree based on the partial 16S rRNA (577 bp) gene sequences was constructed. Multiple sequence alignments were performed using MEGA version 7.0 platform [46]. Sequences were first trimmed at both the 5′ and 3′ ends, so that all sequences start and end at the same nucleotide position. Phylogenetic relationship of the isolates and other Mycobacterium species was investigated in phylogenetic trees constructed using the neighbor-joining method [47]. The neighbour- joining trees were validated using the maximum composite likelihood method and one thousand bootstrap replicates were run. *Nocardia farcinica* was used as an outgroup sequence.

Similarly, neighbor joining trees based on *esxA* and *esxB* gene sequences were constructed using the sequences of *M. marinum* isolates from this study as well as those of other mycobacteria. This was done to determine the position of the isolated *M. marinum* strains in the phylogenetic tree as well as to investigate if phylogeny could give insight into the association of the *esxA* and *esxB* sequences with pathogenicity of mycobacteria as well as growth rate. The *esxA* and *esxB* gene sequences of different *Mycobacterium* species were retrieved from Genbank database (https://www.ncbi.nlm.nih.gov/nucleotide), while the *esxA* and *esxB* sequences of *Mycobacterium nonchromogenicum* NCK 8640 was determined in the previous study describing the whole genome of this isolate [35].

The genetic relationship among the *M. marinum* isolates were inferred from the MIRU-VNTR using the BIONUMERICS software version. 7. 6 (www.appliedmaths.com) applying the unweighted group method with arithmetic average (UPGMA).

### Genbank nucleotide accession numbers

Nucleotide sequences of the following gene fragments: 16S rRNA, *hsp65*, *esxA* and *esxB* for the four *Mycobacterium marinum* strains as well as the 16S rRNA and *hsp65* for *Mycobacterium shimoidei* were submitted to Genbank (www.Ncbi.nlm.nih.gov/Genbank). The accession numbers for the 16Sr RNA genes are, MF402009, MF402010, MF402011, MF402012 and MF402013, for *Mycobacterium shimoidei* strain 1645, *Mycobacterium marinum* strain 1123, *Mycobacterium marinum* strain 526A, *Mycobacterium marinum* strain 6865, and *Mycobacterium marinum* strain 7873 respectively. The accession numbers for *hsp65* are MF411150**,** MF411146, MF411147, MF411148, and MF411149 for *Mycobacterium shimoidei* strain 1645, *Mycobacterium marinum* strain 1123, *Mycobacterium marinum* strain 526A, *Mycobacterium marinum* strain 6865 and *Mycobacterium marinum* and strain 7873 respectively. The accession numbers for *esxA* are, MF411157, MF411153, MF411151 and MF411152 for *Mycobacterium marinum* strain1123, *Mycobacterium marinum* strain 526A, *Mycobacterium marinum* strain 6865 and *Mycobacterium marinum* strain 7873 respectively. The accession numbers for *esxB* are, MF411158, MF411154, MF411155 and MF411156 for *Mycobacterium marinum* strain 1123, *Mycobacterium marinum* strain 526A, *Mycobacterium marinum* strain 6865 and *Mycobacterium marinum* strain 7873 respectively.

## Abbreviations

CFP-10: 10 kDa Culture filtrate protein
ESAT-6: 6 kDa Early secretory antigenic target
NTM: Non tuberculous mycobacteria
PCR: Polymerase Chain Reaction
